# Anticancer Activity of *Vitex agnus-castus* Seed Extract on Gastric Cancer Cells

**DOI:** 10.3390/nu17152564

**Published:** 2025-08-06

**Authors:** Özlem Türksoy-Terzioğlu, Feyza Tosya, Ayşe Büşranur Çelik, Sibel Bölek, Levent Gülüm, Gökhan Terzioğlu, Yusuf Tutar

**Affiliations:** 1 Department of Molecular Biology and Genetics, Hamidiye Institute of Health Sciences, University of Health Sciences-Turkey, Istanbul 34668, Türkiye; ozlem.turksoy@sbu.edu.tr (Ö.T.-T.); ayseclk1899@gmail.com (A.B.Ç.); 2 Experimental Medicine Research and Application Center, University of Health Sciences Turkey, Istanbul 34662, Türkiye; tosyafeyzaft@gmail.com (F.T.); sibel.bolek@sbu.edu.tr (S.B.); 3 Department of Food Technology, Hamidiye Institute of Health Sciences, University of Health Sciences-Turkey, Istanbul 34668, Türkiye; 4 Department of Plant and Animal Production, Mudurnu Süreyya Astarcı Vocational College, Bolu Abant İzzet Baysal University, Bolu 14030, Türkiye; leventgulum@ibu.edu.tr; 5 Department of Medical Biology, Hamidiye International School of Medicine, University of Health Sciences, Istanbul 34899, Türkiye; gokhan.terzioglu@sbu.edu.tr; 6 Department of Basic Medical Sciences, Division of Medical Biochemistry, Faculty of Medicine, Recep Tayyip Erdogan University, Rize 53100, Türkiye

**Keywords:** *Vitex agnus-castus*, gastric cancer, apoptosis, cytotoxicity, epithelial–mesenchymal transition (EMT)

## Abstract

**Background/Objectives**: *Vitex agnus-castus* has been traditionally used to treat hormonal disorders, and recent evidence suggests its potential anticancer properties. However, its effects on gastric cancer remain unclear. **Methods**: This study examined the cytotoxic, apoptotic, and anti-metastatic effects of hydroalcoholic *Vitex agnus-castus* seed extract in gastric cancer cells. Antioxidant capacity (DPPH, ABTS) and total phenolic and flavonoid contents were analyzed. Cytotoxicity was assessed using the MTT assay in HGC27, MKN45, and AGS gastric cancer cell lines and CCD-1072Sk fibroblasts. Apoptosis, mitochondrial membrane potential (MMP), and cell cycle changes were evaluated via Annexin V-FITC/PI, Rhodamine 123, and PI staining, respectively. RT-qPCR and gene enrichment analyses were conducted to investigate the molecular mechanisms. Apoptosis-related protein expression was analyzed through enzyme-linked immunosorbent assay (ELISA). **Results**: The extract exhibited high antioxidant activity and a significant phenolic and flavonoid content. It reduced cell viability in a dose-dependent manner in gastric cancer cells, while exerting low toxicity in fibroblasts. It significantly increased apoptosis, induced G0/G1-phase cell cycle arrest, upregulated pro-apoptotic genes (CASP3, CASP7, TP53, BCL2L11), and downregulated anti-apoptotic genes (XIAP, NOL3). Gene enrichment analysis highlighted pathways like apoptosis, necrosis, and cysteine endopeptidase activity. The extract also disrupted MMP, inhibited migration and spheroid formation, suppressed EMT markers (SNAIL, SLUG, TWIST1, N-CADHERIN), and upregulated E-CADHERIN. The expression of Caspase 3 and Bax proteins increased and Bcl2 protein decreased. **Conclusions**: These findings suggest that Vitex agnus-castus seed extract exerts strong anticancer effects in gastric cancer cells by promoting apoptosis, reducing proliferation, and inhibiting migration. Further studies are warranted to explore its clinical relevance.

## 1. Introduction

The species *Vitex agnus-castus* L., belonging to the Verbenaceae family, is commonly known as vitex, chaste tree, or monk’s pepper and is also referred to as Abraham’s palm, lilac chaste tree, and finger chaste tree. It is characterized by a shrubby growth form, reaching heights between one and five meters, with finger-shaped leaves, blue-violet flowers, and dark purple fruits [1]. The most commonly used part of the plant is the ripe, dried fruit, which is used as extracts or concentrates [2]. *Vitex agnus-castus*, a plant native to the Mediterranean and Central Asia, has traditionally been used to treat various health conditions, including menstrual disorders, infertility, and hormonal imbalances [3]. However, its therapeutic potential extends beyond these traditional uses. Recent studies have begun to reveal its intriguing pharmacological properties, particularly its ability to inhibit cancer cell growth and induce apoptosis in several types of cancer cells [4]. The major components of *Vitex agnus-castus* are identified as casticin, vitexin, isovitexin, apigenin, orientin, isoorientin, luteolin, agnuside, 4-hydroxybenzoic acid, and linoleic acid [3]. *Vitex agnus-castus* seed extract has antioxidant and antibacterial properties due to these components in its structure and is useful for the treatment and improvement of the oxidative state of breast tumor tissue [5,6]. Especially, casticin has anticancer and anti-inflammatory properties via different molecular mechanisms [7]. In recent studies, *Vitex agnus-castus* seed extracts showed significant cytotoxic, DNA damage, and apoptotic effects in MCF-7 breast cancer cells [8]. Studies also showed anti-tumorigenic effects in vitro and in vivo by reducing cyclooxygenase-2 activity and oxidative stress complications [1]. *Vitex agnus-castus* extract inhibited cell proliferation and induced apoptosis in human prostate epithelial cell lines, suggesting potential anticancer effects [9]. Silver nanoparticles synthesized with *Vitex agnus-castus* extract showed potential anti-proliferative effects on HeLa cancer cells with 39.6% ± 7.9% cytotoxicity [10]. Research into *Vitex agnus-castus*’s anticancer effects is still in its early stages but holds promise. Preliminary findings suggest that bioactive compounds found in the plant may interfere with cancer cell signaling pathways, disrupt cell cycle progression, and exhibit antioxidant and anti-inflammatory activities that are beneficial in combating cancer progression [4,8,11]. The objective of this study was to examine the anticancer properties of *Vitex agnus-castus* seed extract on the HGC27 gastric cancer line, to assess the extract’s cytotoxic impact on cancer cells, and to determine its influence on cellular processes such as proliferation, spheroid formation, apoptosis, cell migration, gene expression related to cancer cell signaling pathways, and gene enrichment analysis.

## 2. Materials and Methods

### 2.1. Extraction of Vitex agnus-castus Seeds

The seeds of the *Vitex agnus-castus* plant were obtained from a local market in Pendik, Istanbul, and stored at 20 °C temperature and 60% humidity. All chemicals used were of analytical grade.

The extraction method was based on the method determined by Uslu et al. [12] with some modifications. *Vitex agnus-castus* seeds were ground using a 0.5 mm diameter sieve (Fritsch, Pulverisette 14, Idar-Oberstein, Germany). Then, 60 g of ground seed powders were added to 300 mL of 50% ethanol *v*/*v* (50% ethanol:50% ultra-pure water), vortexed for 3 min, and filtered with Whatman No.4. Ethyl alcohol in hydroalcoholic solution was evaporated at 105 mbar, 32 °C, at a rotation speed of 50 RPM. The remaining aqueous *Vitex agnus-castus* seed extract was lyophilized (BUCHI L200, Flewil, Switzerland) at −56 °C and 0.1 mbar for 24 h. The powder extract was dissolved in 0.01% DMSO before application to cells and filtered through a sterile filter with a pore diameter of 0.22 µm.

### 2.2. Antioxidant Activity of Vitex agnus-castus Seeds’ Extract

#### 2.2.1. DPPH Assay

The antioxidant activity of *Vitex agnus-castus* extract was determined by DPPH radical using the method of Brand-Williams et al. [13] with slight modifications. Briefly, 600 μL of 0.1 mM DPPH reagent and 20, 40, 60, 80, and 100 µL of methanolic extract were added to 6 mL of methanol in a tube and kept in the dark for 20 min. The absorbance was measured at 517 nm. IC50 value was calculated by constructing a concentration-dependent inhibition change curve.

#### 2.2.2. ABTS Assay

ABTS radical scavenging activity was determined according to the method described by Re et al. [14]. Briefly, it is based on the formation of the free radical cation ABTS in a reaction between ABTS (7 mM) and K_2_S_2_O_8_ (2.45 mM) at RT, in the dark, for 12–16 h. Then, 10, 20, and 30 μL volumes of methanolic extract were added to 1 mL of 2% ABTS solution, and absorbance was measured at 734 nm. The slope of the concentration curve determined with 3 different concentrations was converted to Trolox equivalence.

### 2.3. Total Phenolic Content of Vitex agnus-castus Seeds’ Extract

Total phenolic content was determined according to the method of Singleton and Rossi [15] with slight modifications. The powdered *Vitex agnus-castus* seed extract was diluted 1:10 with methanol. Then, 500 μL of Folin–Ciocalteu reagent and 100 μL of methanolic extract were added to 7.5 mL of distilled water and kept for 3 min. A total of 1 mL of supersaturated sodium bicarbonate solution was added, and the total volume was added to 10 mL of distilled water. Absorbances of samples were measured using a spectrophotometer (Thermo Scientific, Multiskan-Sky, Waltham, MA, USA) at a wavelength of 720 nm, and the total phenolic content was expressed as mg gallic acid equivalence/100 g. Analyses were performed in 3 replications.

### 2.4. Total Flavonoid Content of Vitex agnus-castus Seeds’ Extract

Total flavonoid content of the extract was performed according to the method described by Amorim et al. [16]. Briefly, 500 μL of 60% (*v*/*v*) acetic acid solution, 2 mL of 20% (*v*/*v*) pyridine, 1 mL of 5% (*w*/*v*) AlCl_3_, and 6 mL of 80% (*v*/*v*) methanol were added, respectively, to 500 μL of extract at a concentration of 1 mg/mL dissolved in 80% *(v*/*v*) methanol. The prepared solution was kept at room temperature for 30 min. Absorbance at 420 nm was measured. The results were determined as mg rutin equivalence/100 g of sample.

### 2.5. Cell Culture and MTT Cytotoxicity Assay

HGC27 cell line (Accession CVCL_1279), derived from the metastatic lymph node of gastric cancer, MKN45 cell line (Accession CVCL_0434), derived from the liver metastasis of a poorly differentiated gastric adenocarcinoma, and AGS epithelial tumorigenic cell line (Accession CVCL_0139), originated from the stomach tissue of a patient with gastric adenocarcinoma, were kindly provided by Prof. Dr. Omer Faruk Bayrak and maintained in RPMI-1640 medium (Thermo Fisher Scientific, Waltham, MA, USA). CCD-1072Sk cell line (CRL-2088, ATCC; Accession CVCL_2333), which was established from skin taken from normal foreskin, was kindly provided by the cell repository of Yıldız Technical University and cultured in DMEM-F12 medium (Capricorn, Ebsdorfergrund, Germany) containing 10% FBS, with 100 units/mL penicillin and 100 g/mL streptomycin (Thermo Fisher Scientific, Waltham, MA, USA).

The MTT (3-[4,5-dimethylthiazol-2-yl]-2,5 diphenyl tetrazolium bromide) assay provides insight into cytotoxicity in cells through the assessment of the conversion of MTT into formazan crystals, which determines mitochondrial activity.

Cells were seeded in 96-well plates at a density of 2000 cells/well in 100 μL of RPMI-1640 medium (3 wells per group) with or without Vitex agnus-castus extract. For the cytotoxicity assay, the cells were treated with 2.5–100 µg/mL of Vitex agnus-castus and DMSO (0.01%) for 48 h. MTT assay was used to determine the relative survival fraction of cells. Then, 20 µL MTT ((3-[4,5-dimethylthiazol-2-yl]-2,5 diphenyl tetrazolium bromide) reagent and 80 µL growth medium were added to each well at the 48th hour of extract treatment and incubated at 37 °C for 2 h. Then, the medium was aspirated, and 100 µL DMSO was added. Cell viability was assessed according to the absorbance measurements at 570 nm with an ELISA microplate reader (Biotek Synergy H1). Half-maximal inhibitory concentration (IC50) which is the concentration of compounds required to decrease cell viability by 50%, was found out by three independent experiments, each in triplicate. We plotted the percentages calculated for the different sample concentrations and estimated the mean cytotoxic concentration values (IC50) by a linear regression analysis [17].

### 2.6. Spheroid Formation Assay

Spheroid bodies were derived by plating 4 × 10^4^ cells in 24-well plates coated with 2% agarose. HGC27 and CCD-1072Sk cells were cultured in RPMI-1640 medium with 100 units/mL penicillin and 100 g/mL streptomycin (Thermo Fisher Scientific, Waltham, MA, USA), without FBS. After the 10th day of the culture, spheroid bodies were counted under an inverted microscope (Leica, Wetzlar, HE, Germany) at 100× magnification [18].

### 2.7. RNA Extraction and Real-Time Quantitative Polymerase Chain Reaction (RT-qPCR) and Gene Enrichment Analysis

HGC27 cells were seeded into 6-well plates at a density of 4.5 × 105 cells per well and incubated for 48 h. After the incubation period, IC50 dose of extract was applied, and 48 h later, the cells were collected by trypsinization. Following centrifugation at 400× *g* for 5 min, the supernatant was discarded, and the pellet was stored at –80 °C. RNA isolation was performed using an innuPREP RNA Mini Kit 2.0 (IST Innuscreen GmbH, Berlin, Germany). A cDNA synthesis was carried out using a Wonder RT-cDNA Kit (Euroclone, Pero, Italy). A total of 23 cancer progression-related genes, as listed in Table 1, were selected and analyzed using a FluoCycleII SYBR Master Mix Kit (Euroclone, Pero, Italy). ACTINB was used as reference gene, and gene expression changes were calculated using the Livak Method.

The genes that showed significant changes in expression were visualized using the Gene Ontology database in the SRplot program [19].

### 2.8. Apoptosis Assay

Apoptosis analysis was conducted using an Annexin V-FITC/PI Apoptosis Kit (Elabscience, Houston, TX, USA) following the manufacturer’s instructions. A total of 8 × 10^5^ cells per well were seeded into 6-well plates, and extract treatment was applied after 24 h. After 48 h of incubation, the media from the wells were collected into centrifuge tubes, and the wells were washed with PBS, which was also transferred into the same tubes. The cells were detached using trypsin and added to the tubes, followed by centrifugation at 300× *g* for 5 min. The supernatant was discarded, and the pellet was washed twice with cold PBS, with centrifugation at 300× *g* for 5 min each time. The pellet was resuspended in 500 µL of 1× Annexin V Binding Buffer, and 5 µL each of Annexin V-FITC and PI were added. The tubes were vortexed and incubated for 20 min in the dark. Analysis was performed within 1 h using a CytoFLEX Flow Cytometry (Beckman Coulter, Brea, CA, USA).

### 2.9. Wound Healing Assay

After HGC27 cells were seeded onto 6-well plates, we incubated them until they reached 85% confluency. First, the media in the plate were aspirated, and the cells were washed with 1 mL PBS. A scratch was made on the bottom of the plates with a pipette tip. Media containing 1% FBS and extract with an IC50 value were added. DMSO (0.01%) was applied instead of extract to the control well. In this way, 0, 24, and 48 h images were obtained for both drug and control cells using an inverted microscope Paula (Leica, Wetzlar, HE, Germany). Then, we analyzed and compared the gap closure through the wound healing tool of Paula Cell Imager, Leica [20].

### 2.10. Analysis of Mitochondrial Membrane Potential by Rhodamine 123 Staining

We used Rhodamine 123, a specific type of fluorescent dye, to observe the alterations in mitochondrial membrane potential, which is important as an indicator of mitochondrial function. HGC27 cells were seeded into 6-well plates at a density of 4.5 × 10^5^ cells per well and incubated for 48 h. After the incubation period, IC50 dose of extract was applied, and 48 h later, the medium was aspirated, washed with phosphate-buffered saline (PBS), and then stained with 1 µg/mL of Rhodamine 123 (R8004, Sigma-Aldrich, Darmstadt, HE, Germany). After 10 min of incubation at 37 °C, we washed the cells three times with PBS and then added 400 µL of complete medium to prevent them from drying out. We then observed the cells with a fluorescence microscope in both bright and green lights. Finally, we used Image J (version 1.53q) to analyze the fluorescence intensity [21,22].

### 2.11. Cell Cycle Analysis

For cell cycle analysis, cells were first harvested and washed with phosphate-buffered saline (PBS). Fixation was carried out by slowly adding cold ethanol to the cell suspension while vortexing, reaching a final ethanol concentration of 70%. The fixed cells were kept on ice for a minimum of two hours to ensure adequate permeabilization. Following fixation, the cells were washed once with PBS and subsequently resuspended in a staining solution containing PBS supplemented with 100 μg/mL RNase A and 50 μg/mL propidium iodide. To enhance membrane permeability, 0.1% Triton X-100 was included in the staining buffer. All steps involving propidium iodide were performed in the dark. The cells were incubated in the staining solution overnight at 4 °C. DNA content was then analyzed using the CytoFLEX Flow Cytometry (Beckman Coulter, Brea, CA, USA) [23].

### 2.12. Determination of Apoptotic Protein Levels by ELISA

HGC27 cells (5 × 10^6^) were cultured in T-75 flasks for 24 h, and then the culture medium was replaced either with RPMI alone (serving as the untreated control) or RPMI supplemented with the IC50 value of the extract. Cells were incubated under these conditions for 48 h. Subsequently, proteins were extracted and their concentrations were quantified using the BCA protein assay. The expression levels of key apoptotic proteins—BAX, BCL-2, and active caspase 3—were measured with an ELISA microplate reader (Biotek Synergy H1) using the following commercial kits according to the manufacturers’ protocols: human BAX ELISA kit (E-EL-H0562, Elabscience, Houston, TX, USA), human BCL-2-like Protein ELISA kit (E-EL-H0562, Elabscience, Houston, TX, USA), and human Caspase-3 ELISA kit (E-EL-H0562, Elabscience, Houston, TX, USA) [24].

### 2.13. Statistical Analysis

The Kruskal–Wallis and Mann–Whitney U tests were used for statistical analysis. The differences were considered statistically significant at *p* < 0.05 (indicated as ‘*’).

## 3. Results

### 3.1. Phytochemical Composition and Antioxidant Properties of Vitex Agnus-Castus Extract

The total flavonoid content of the *Vitex agnus-castus* seed extract was found to be 5765.86 ± 74.35, and the total phenolic content was found to be 1700.77 ± 163.37 (Table 2). Antioxidant activity was determined as 68.19 ± 4.68% inhibition (IC_50_) for the DPPH method and 323.529 ± 0.33 for the ABTS method. The Vitex extract was shown to have scavenging properties of DPPH radicals and cationic ABTS radicals. A color change was observed in the total antioxidant activity test by scavenging ABTS cationic radicals causing discoloration of the reaction mixture. The tested extract demonstrated an increase in cationic scavenging ability, as observed according to standards.

### 3.2. Cytotoxic Activity of the Vitex Agnus-Castus Extracts on HGC27, MKN45, AGS, and CCD-1072Sk Cells

**Figure 1:**
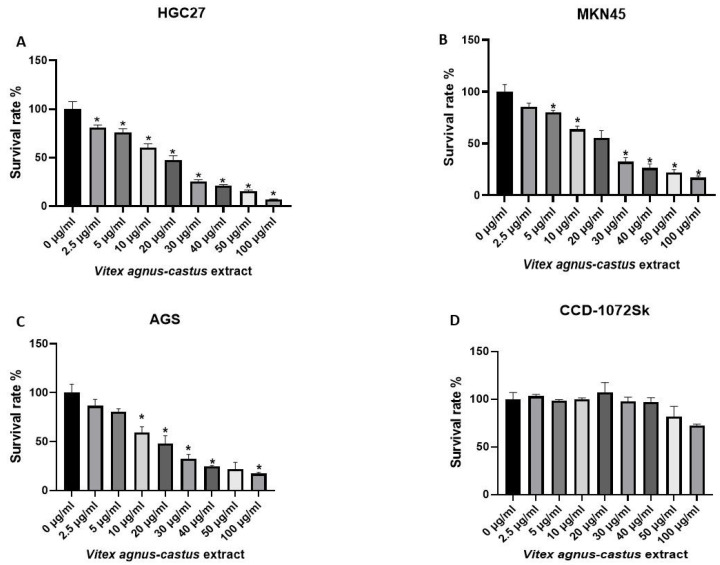
Comparison of cytotoxicity between control and *Vitex agnus-castus* extract treatment for 48 h in HGC27, MKN45, AGS, and CCD-1072Sk cells. The extract significantly inhibited cell survival in dose-dependent manner in (**A**) HGC27, (**B**) MKN45, and (**C**) AGS gastric cancer cells. (**D**) The extract decreased cell survival at higher doses (50 µg/mL and higher concentrations) in CCD-1072Sk cells. The ‘*’ indicates a statistically significant difference between control and treatment groups (*p* < 0.05).

The cytotoxic effects (IC50) of the extract on HGC27, HGC27, MKN45, and AGS cells are shown in Figure 1. We treated the cells with various concentrations of the extracts for 48 h and DMSO (0.01%). We found out that cell survival rates were decreased compared with the control according to the MTT assay. The Vitex extract led to a significant inhibition of cell viability. The cytotoxic effect of the extracts was linearly correlated with the extract concentration (*p* < 0.05). The IC50 value was 26.59886 μg/mL for HGC27, 33.03741 μg/mL for MKN45, and 31.44794 μg/mL for AGS cells.

However, the IC50 value was 176.514 μg/mL for CCD-1072-SK cells at the 48th hour. The Vitex extract did not have a significant cytotoxic effect on human fibroblast cells. However, it exhibited a slightly supportive effect on the proliferation of CCD-1072-SK cells at 2.5 µg/mL, 10 µg/mL, and 20 µg/mL doses of the extract (Figure 1). The selectivity index (SI) was 6.636 for HGC27, 5.342 for MKN45, and 5.612 for AGS cells. SI > 1.0 indicates that a tested compound shows higher selectivity against cancer cells than cytotoxicity against healthy cells [25].

### 3.3. The Effect of the Vitex Agnus-Castus Extract on Apoptosis and Cell Cycle Regulation of HGC27 Cells

**Figure 2:**
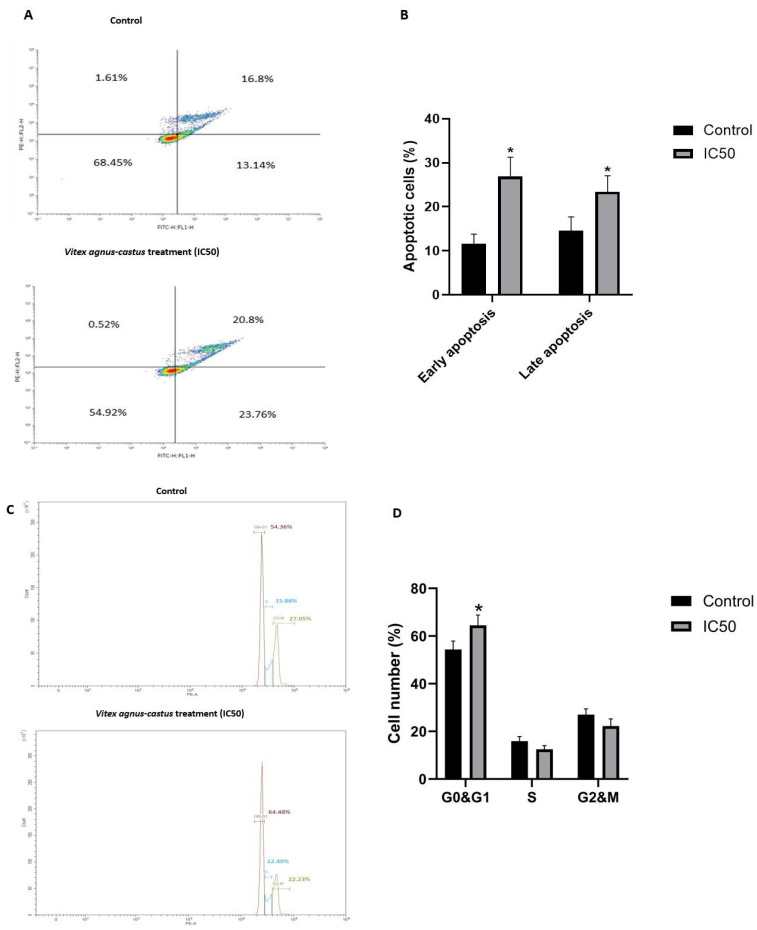
Assessment of apoptosis in HGC-27 cells treated with Vitex agnus-castus extract using Annexin V-FITC/PI double staining. Cells were treated with IC50 dose of the extract for 48 h, with DMSO-treated cells serving as the control group. (**A**) Representative flow cytometry dot plots illustrate the distribution of apoptotic populations. In each plot, viable cells appear in the lower left quadrant (Annexin V−/PI−); early apoptotic cells, in the lower right quadrant (Annexin V+/PI−); and late apoptotic or necrotic cells, in the upper right quadrant (Annexin V+/PI+). (**B**) Bar graphs represent the quantitative analysis of cells in different apoptotic stages based on flow cytometry data. (**C**) The effect of the Vitex extract on the cell cycle in HGC27 cells, leading to cell cycle arrest in the G0/G1 phase. (**D**) Bar graphs represent the quantitative analysis of cells in different cell cycle phases based on flow cytometry data. The ‘*’ indicates a statistically significant difference between control and treatment groups (*p* < 0.05).

We treated HGC27 cells with the IC50 value of the extract, and it induced apoptosis significantly after 48 h (Figure 2).

**Figure 3:**
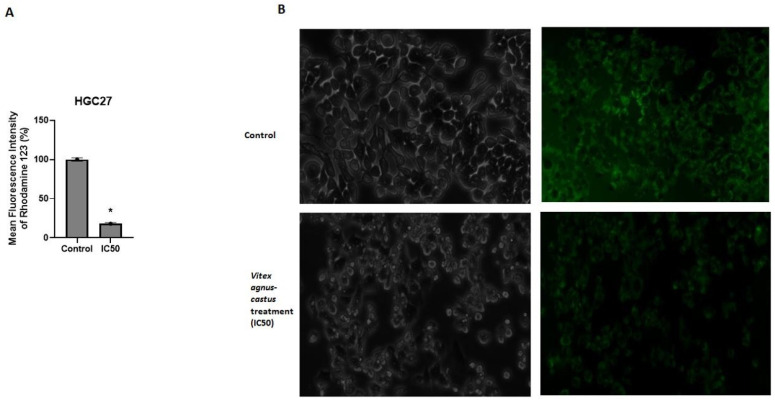
Measurement of mitochondrial membrane potential using Rhodamine 123 following 48 h of *Vitex agnus-castus* treatment. (**A**) Microscopic images, (**B**) mean fluorescence intensity of HGC27 cells. The ‘*’ indicates a statistically significant difference between control and treatment groups (*p* < 0.05).

Rhodamine 123 staining demonstrated that mitochondrial membrane potential was significantly reduced in HGC27 cells upon extract (IC50) treatment (*p* = 0.0421) (Figure 3A,B). It was also shown that the extract (IC50) induced G0/G1-phase cell cycle arrest. Extract treatment increased the proportion of cells in the G0/G1 phase (64.48%) compared to the control group (54.36%) (*p* = 0.0261). Extract treatment slightly decreased the proportion of the cells in the S phase and G2/M phase compared to the untreated control group (Figure 2).

### 3.4. The Extract Treatment Decreased the Spheroid Formation and Migration Capacity of HGC27 Cells

**Figure 4:**
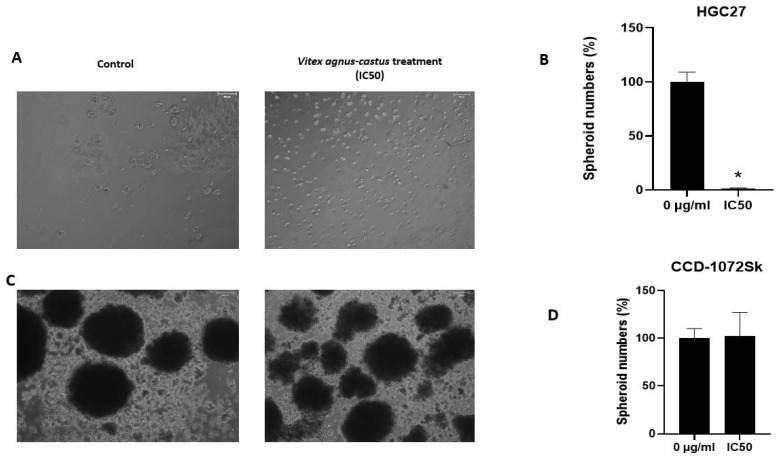
Effect of IC50 value of *Vitex agnus-castus* extract on spheroid formation capacity of HGC27 and CCD-1072Sk. Images were acquired 10 days after seeding and treatment using PAULA Cell Imager. (**A**,**C**) The spheroid formation capacity HGC-27 and CCD-1072Sk, respectively: left panel shows control, right panel shows the effect of Vitex treatment. (**B**,**D**) Spheroid numbers for HGC-27 and CCD-1072Sk, respectively. Scale bar 100 µm; inverted microscopy at 100× magnification. Data are presented as the means ± SD. Comparison of spheroid-forming activity. The ‘*’ indicates a statistically significant difference between control and treatment groups (*p* < 0.05).

The spheroid culture system is used for the enrichment of cancer stem cells (CSCs) [18]. It is a valuable method to detect the effect of the tested compound on cancer stem cell formation. The IC50 value of the extract significantly decreased spheroid body formation in HGC27 cells (*p* = 0.008562) (Figure 4A,B). However, IC50 value of the extract slightly increased spheroid body formation in CCD-1072Sk cells (Figure 4C,D).

Cell migration is crucial for metastasis. The migratory capacity of cells affects patient prognosis and survival, as metastasis is responsible for more than 90% of cancer deaths [26].

**Figure 5:**
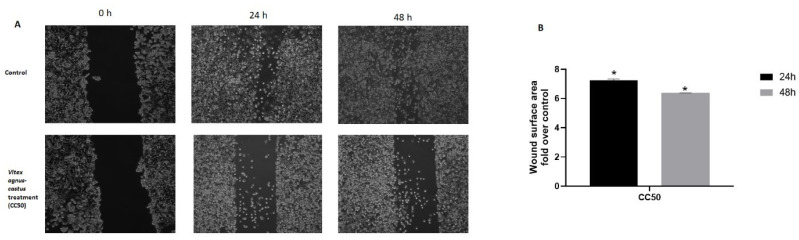
Effect of IC50 dose of *Vitex agnus-castus* extract on HGC27 migration (scratch test). (**A**) Images from the top panel show control; bottom panel shows treated cells. Images were acquired 0, 24, and 48 h after scratching and treatment using a PAULA Cell Imager. PAULA Cell Imager’s wound healing software was used to analyze the images. (**B**) Wound surface area as fold over control at the 24th and 48th hours. Scale bar 100 µm; inverted microscopy at 100× magnification. The ‘*’ indicates a statistically significant difference between control and treatment groups (*p* < 0.05).

We performed a wound healing assay to observe the effect of the extract (IC50) on the migratory capacity of HGC27 cells. After administration of the extract for 24 h and 48 h, the treated group had a 7.24- and 6.38-fold higher wound surface area compared to control, respectively (Figure 5).

### 3.5. The Extract Treatment-Altered Gene and Protein Expression in HGC27 Cells

**Figure 6:**
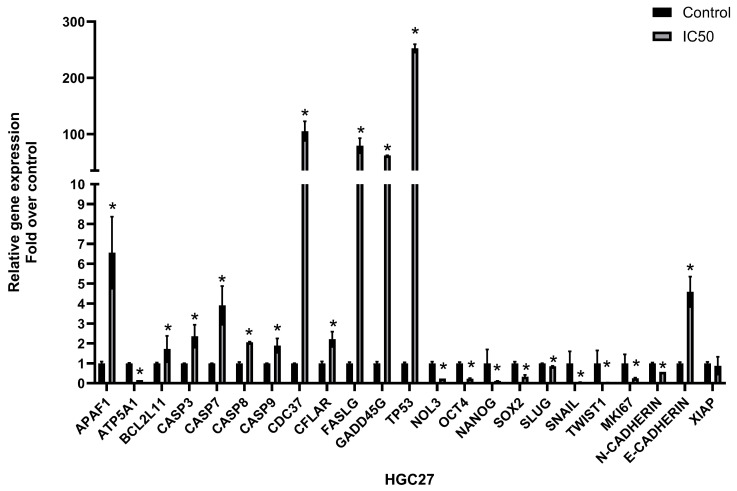
Effect of IC50 dose of *Vitex agnus-castus* extract on the gene expression pattern related to cancer progression of HGC27. Fold over control at 48th hour. mRNA levels were normalized with respect to ACTINB. The ‘*’ indicates a statistically significant difference between control and treatment groups (*p* < 0.05).

The expression of cancer progression-related genes was analyzed by qRT-PCR to determine whether the extract treatment caused alteration in the gene expression of HGC27 cells. The results indicated that the IC50 dose of the extract led to different expression patterns of stem cell-, apoptosis-, epithelial-, and mesenchymal-related markers. The extract treatment significantly decreased the expression of stem cell-related markers including OCT4, NANOG, and SOX2 (*p* = 0.0006, *p* < 0.00001, and *p* = 0.004, respectively) and mesenchymal markers including SLUG, SNAIL, TWIST1, and N-CADHERIN (*p* = 0.008, *p* = 0.00002, *p* < 0.00001, and *p* = 0.00003, respectively). The expression of proliferation, epithelial, and mitochondrial ATP synthase-related markers including MKI67, E-CADHERIN, and ATP5A1 was increased upon extract treatment (*p* = 0.00001, *p* = 0.01, and *p* < 0.00001, respectively). The expression of the markers related to apoptosis induction including APAF1, BCL2L11, CASP3, CASP7, CASP8, CDC37, CFLAR, FASLG, GADD45G, and TP53 was increased (*p* = 0.08, *p* = 0.02, *p* = 0.03, *p* = 0.02, *p* = 0.006, *p* = 0.02, *p* = 0.007, *p* < 0.00001, and *p* = 0.0002, respectively). The expression of the anti-apoptotic genes’ markers including XIAP and NOL3 was decreased (*p* = 0.37 and *p* < 0.00001). Gene expression changes are shown in Figure 6.

**Figure 7:**
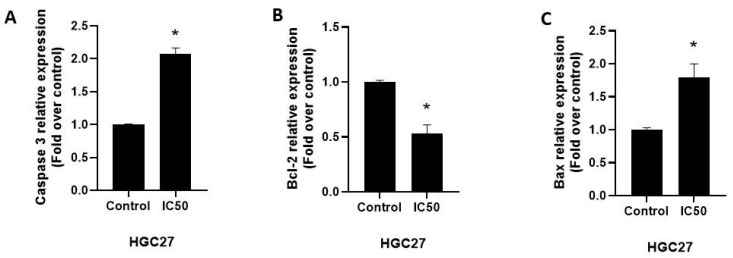
Effect of IC50 dose of *Vitex agnus-castus* extract on the Caspase3, Bcl-2, and Bax protein expressions in HGC27 cells. Relative expression of (**A**) Caspase 3, (**B**) Bcl-2, and (**C**) Bax proteins. The ‘*’ indicates a statistically significant difference between control and treatment groups (*p* < 0.05).

The expression of apoptosis-related proteins was detected by ELISA. Consistent with the gene expression results, the IC50 dose of the extract significantly increased the expression of pro-apoptotic protein Bax and effector protein Caspase3 and decreased the expression of anti-apoptotic protein Bcl-2 (Figure 7).

**Figure 8:**
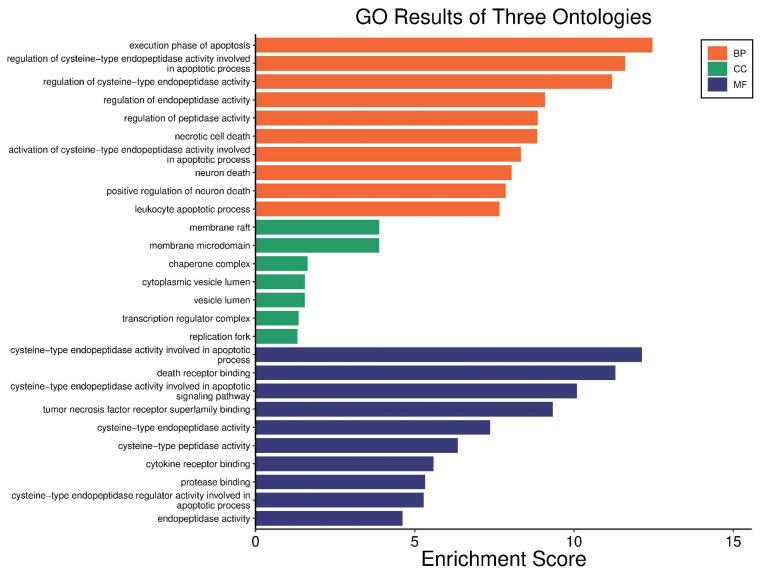
Gene enrichment analysis was performed using the Gene Ontology (BP, CC, MF) database. Among the programmed cell deaths, apoptosis and apoptosis-related cellular components were observed. The analysis and visualizations were conducted using SRplot; https://www.bioinformatics.com.cn/en (accessed on 23 April 2025).

**Figure 9:**
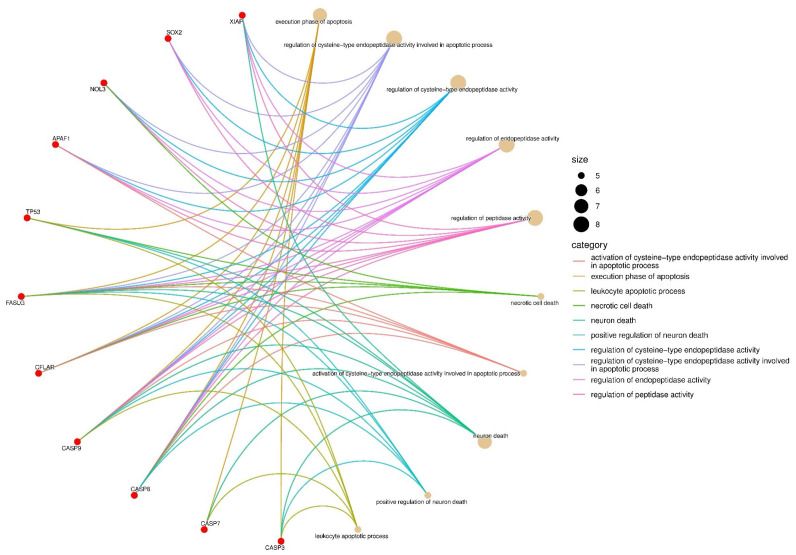
Gene ontology biological process analysis was performed to identify gene-to-cellular event associations https://www.bioinformatics.com.cn/en (accessed on 23 April 2025).

The genes with observed expression changes were analyzed using the Gene Ontology database. Ontologies related to biological process (BP), cellular component (CC), and molecular function (MF) were identified (Figure 8). In the BP analysis, gene-to-cellular event associations were mapped (Figure 9). In all three gene ontology analyses, the formation and regulation of apoptosis and necrosis were observed.

## 4. Discussion

Antioxidant properties of phytochemicals can be determined by several mechanisms including radical scavenging, ion or metal coupling, peroxide decomposition, and reduction. These properties suppress oxidative stress, which causes food spoilage and diseases such as atherosclerosis, cancer, and Alzheimer’s [1]. *Vitex agnus-castus* seed extracts have antioxidant properties [1,8]. Ibrahim et al. [1] found that *Vitex agnus-castus* seed extracts scavenged radicals and hydrogen peroxide. Another research item supports the antioxidant potential of *Vitex agnus-castus* extracts, which scavenged different radicals and showed the ability to scavenge hydrogen peroxide [27]. In a study in which the antioxidant and anticancer activity of *Vitex agnus-castus* seeds was evaluated by extracting them with different solvents, methanol and water extracts had significant DPPH scavenging activity and all of the extracts had significant cytotoxic, DNA damaging, and apoptotic effects in MCF-7 human breast cancer cells [8]. We demonstrated a significant antioxidant activity of *Vitex agnus-castus* seed extracts through the DPPH and ABTS methods. The extract also had a high total phenolic and flavonoid content, and this may contribute to its antioxidant activities and capacity to scavenge free radicals [28]. Vitex extract showed anti-proliferative effects on HGC27, MKN45, and AGS gastric cancer cells in a dose-dependent manner (Figure 1). Phenolic compounds can alter the redox balance of cancer cells by becoming pro-oxidants [29]. The potential cytotoxic effect observed in this study may be attributed to the phenolic compounds found in the extract. On the contrary, it did not significantly affect the viability and spheroid formation capacity of healthy fibroblast cells. Anticancer agents with cytotoxic properties in cancer cells and without showing cytotoxicity on healthy cells are crucial for developing new therapeutics against cancer.

The effect of the seed extract on gastric cancer cells was investigated in this study. The apoptotic effects of Vitex extract treatment were shown in several studies with diverse cell lines of several cancer types through different cellular mechanisms of the apoptosis induction [4,11,30].

Vitex extract treatment stimulated caspase-9 and -3 caused by ER stress in KATO-III, a human gastric signet ring carcinoma cell line. The authors suggested that Vitex-induced apoptosis was contributed predominantly by JNK, followed by the activation of caspase family molecules. Caspase-8 was activated in the Vitex-treated KATO-III; however, the activation did not occur in Vitex-treated COLO 201 [11]. In this study, it was demonstrated that the extract of the seed of Vitex stimulated apoptosis in the HGC27 gastric cancer cell line, which is consistent with the study of Ohyama et al. [11] performed on KATO-III [11]. It was demonstrated that luteolin, which was a component of Vitex and essential oil of Vitex, stimulated apoptosis through the mitochondrial pathway in HepG2 and A549 cells, respectively [30,31]. Additionally, Vitexins, which were derived from Vitex negundo with flavonoid, was demonstrated to have apoptotic effects against ovarian, breast, and prostate cancer cells by an intrinsic pathway [32]. Essential oils derived from *Vitex agnus-castus* led to caspase 3/7 activation and an increase in apoptosis through stimulating both extrinsic and intrinsic pathways by modulating Bax, Bad, Bcl-2, Bcl-XL, FADD, Caspase-8, Caspase-9, TRAIL R1/DR4, and TRAIL R2/DR5 [33].

It seems that different mechanisms had a function in the apoptosis induction of different types of cancer cells upon Vitex treatment. Our results showed an increase in the levels of mRNAs of pro-apoptotic genes that were analyzed in this study, APAF-1, BID, BCL2L11, FASLG, GADD45G, CASP8, CASP3, CASP7, CASP9, and TP53, together with an increase in apoptosis and the apoptotic protein markers (BAX and caspase 3). A decrease in the expression of the anti-apoptotic genes, NOL3 and XIAP, and in the anti-apoptotic protein marker, BCL-2, was observed upon extract treatment. It was suggested that the mitochondrial apoptosis pathway with the activation of APAF-1; Caspase-3, -7, and -9; and Bax and the downregulation of Bcl-2 was involved in the apoptosis induction in HGC27 upon Vitex treatment that is consistent with the studies mentioned above [30,31,32,34].

BCL2L11 is a well-known gene that functions to mediate cell apoptosis. BCL2L11 functions as a tumor-suppressor gene in diverse types of cancers, such as gastric cancer, lymphoma, and rectal cancer [35]. It was demonstrated that the expression of BCL2L11 decreased in gastric cancer tumor tissues by nearly 70% of that in para-carcinoma tissues [36]. TP53 is another important tumor suppressor gene that has functions in apoptosis, cell cycle, DNA repair, etc. [37]. The TP53-encoded p53 protein induces apoptosis through upregulating apoptosis-related proteins [37]. Caspase 9 functions as an initiator caspase in the intrinsic or mitochondrial caspase pathway and plays a significant role in the stimulation of the caspase cascade, which functions in apoptosis activation. Caspase-9 binds to Apaf-1, a human homolog of the Caenorhabditis elegans CED4 protein, to form the apoptosome, which consists of cytochrome c, Apaf-1, and caspase-9. It cleaves and stimulates effector caspases, caspase-3 (CASP3) or caspase-7 (CASP7), inducing apoptosis in an ABL1/c-Abl-dependent manner [38,39]. The X-linked mammalian inhibitor of apoptosis protein (XIAP) has anti-apoptotic functions through the inhibition of caspases 3, 7, and 9 [40]. The NOL3 gene encodes nucleolar protein 3 (also known as apoptosis repressor with caspase recruitment domain, ARC). ARC is known to have the ability to block cell death by antagonizing the intrinsic and extrinsic apoptosis pathways [41].

The increase in the expression of the APAF-1, BCL2L11, CASP3, CASP7, CASP9, and TP53 genes and the decrease in the expression of XIAP and NOL3 upon Vitex treatment showed that the intrinsic pathway was potentially activated in HGC27 cells. Rhodamine 123 staining also showed that mitochondrial membrane potential (MMP) was decreased in HGC27 upon Vitex treatment; a fall in the mitochondrial membrane potential was known to stimulate mechanisms of apoptosis [42]. This result is also consistent with the results of the gene expression, annexin V/PI assay, and apoptosis-related protein expression analysis. Decreased MMP is one of the key events during apoptosis [43,44]. The impact of the extract on cell cycle progression was also investigated in HGC27 cells. Proper regulation of the cell cycle is essential for maintaining controlled cellular proliferation, and its disruption is a hallmark of cancer [45]. The treatment resulted in a significant increase in the percentage of cells arrested in the G0/G1 phase when compared to the untreated control. To strengthen these findings, future research should focus on analyzing the expression of key regulators involved in the G0/G1 transition, such as the p21 and p16 pathways, to better understand the molecular mechanisms contributing to the observed G0/G1 arrest [46].

Vitex treatment increased the expression of CASP8 and FASLG. This shows an engagement between the extrinsic pathway and the intrinsic pathway. BID expression, which is stimulated after caspase-8-mediated cleavage, establishes the link between these pathways and Vitex-induced BID expression. The dual activation of extrinsic and intrinsic apoptosis pathways may lead to the effective induction of cell death [47].

In gene ontology and gene enrichment analyses, the activation of cysteine-type endonucleases and subsequent apoptosis were observed. The cathepsin family consists of proteolytic enzymes, including serine, aspartate, and cysteine proteases [48]. Cysteine cathepsins are involved in various intracellular events such as extracellular matrix (ECM) remodeling, apoptosis, and cell cycle regulation [49]. It is known that cysteine cathepsins play a role in the early stages of apoptosis by mediating the cleavage of procaspases, the release of pro-apoptotic factors such as cytochrome c, the degradation of anti-apoptotic BCL-2 family members, and the degradation of XIAP, thereby promoting apoptotic progression [50,51]. In our RT-qPCR results, the observed downregulation of XIAP expression, together with the activation of caspase-9, which is directly inhibited by XIAP, and other downstream caspases, suggests the triggering of the intrinsic apoptotic pathway. The increased expression of caspases 3, 7, 8, and 9, along with decreased XIAP expression, may indicate that this process is driven by the activation of cysteine cathepsins.

Vitex treatment decreased the expression of ATP5A1, which is an important gene that encodes for a subunit of ATP synthase (complex V). Its excessive representation had a function in an oxidative phosphorylation pathway that is related to tumor progression [52]. A current study showed that a higher expression level of ATP5A1 was associated with a poor long-term prognosis of gastric cancer patients and it functions partly through inducing cell glucose metabolism [53]. We showed that Vitex treatment decreased the gene expression level of ATP5A1 in HGC27 cells, which may alleviate poor prognostic characteristics in gastric cancer cells.

GADD45G is involved in the DNA damage-inducible gene family. GADD45G blocks cell growth and induces apoptosis in stress shock. Its transcriptional downregulation or silencing was frequently shown in tumor cells including Hodgkin’s and non-Hodgkin’s lymphoma, nasopharyngeal carcinoma, cervical carcinoma, esophageal carcinoma, lung carcinoma, gastric adenocarcinoma, and other tumor types. The ectopic expression of GADD45G was shown to inhibit colony formation and tumor cell growth in silenced cell lines. It was suggested that GADD45G may function as a tumor suppressor, however frequently inactivated epigenetically in several cancer types [54,55]. The expression of GADD45G strongly increased upon Vitex treatment in HGC27 cells. This result is consistent with the decrease in proliferation and spheroid formation capacity. The molecular mechanisms related to the increased GADD45G expression upon Vitex-induced stress deserve further investigation.

The expression of genes related to cancer stemness and the epithelial–mesenchymal transition were analyzed by RT-PCR. Cancer stem cells (CSCs) are a small group that consists of cells that resemble stem cells with self-renewal capability, plasticity, and potency. They can be responsible for tumor initiation and drug resistance [56]. The spheroid culture method is a classical method to enrich CSCs in vitro and mimics the in vivo conditions [57,58]. NANOG, SOX2, and OCT4 are highly critical stemness genes that promote cancer stem cell (CSCs)-related characteristics including the self-renewal of CSCs [59]. NANOG, SOX2, and OCT4 were found to be highly expressed in gastric cancer tumor tissues compared with those in adjacent healthy tissues, and their overexpression was associated with tumor size, tumor grade, and overall survival time [60]. At IC50 doses, the extract was shown to decrease the expression of NANOG, SOX2, and OCT4 genes together with a significant decrease in the spheroid formation capacity in HGC27 cells.

Epithelial-to-mesenchymal transition (EMT) is a fundamental process in embryonic morphogenesis and is known to have crucial function in the progression of primary tumors toward metastasis [61]. Upon EMT induction, cells go through a development-related switch from a polarized epithelial phenotype to mesenchymal phenotype with high motility [62].

SNAIL, SLUG, TWIST1, N-CADHERIN (neural cadherin), and E-CADHERIN genes are important regulators of EMT. Loss of E-CADHERIN expression is one of the most important factors for EMT initiation because its expression is crucial for an epithelial phenotype through the maintenance of the stability of adherends junctions; a decrease in its expression is related to progression and metastasis in various types of cancer [62,63].

Slug and Snail are zinc finger proteins that are responsible for apoptosis resistance, cell motility, and tumor progression. They trigger a decrease in the E-CADHERIN expression via interaction with E-cadherin promoter [64]. Another important molecule, Twist1, is a conserved basic helix-loop-helix transcriptional factor that is known to activate N-cadherin, which is related to tumor invasiveness; the increased expression of Twist1 is known to be related to tumor progression, invasion, and drug resistance [65,66]. In this study, it was observed that the mRNA expression of SNAIL, SLUG, TWIST1, and N-CADHERIN was decreased and the expression of E-CADHERIN was increased upon IC50 doses of extract treatment. Along with this, extract treatment significantly inhibited cell motility, which is consistent with the results of the gene expression.

## 5. Conclusions

In conclusion, the extract from seeds of *Vitex agnus-castus* L. had a high cytotoxic activity on gastric cancer cells, while there was no cytotoxic activity on human fibroblast cells. At IC50 doses, the extract was shown to induce apoptosis, disrupt the MMP, and decrease the capability of migration and spheroid formation in the HGC27 cells. Consistently, the extract altered the expression of the genes related to apoptosis, stemness, and the epithelial–mesenchymal transition. Anti-proliferative and apoptotic effects of the Vitex extract against gastric cancer cells may be attributed to their antioxidant activity and high phenolic contents. Bioactive content with anticancer activity and mechanisms of antioxidant and anticancer activities needs further investigation. This study gives a scientific rationale for utilizing *Vitex agnus-castus* L. in the future development of therapeutics against gastric cancer.

## Figures and Tables

**Table 1:** Cancer progression-related genes analyzed in the study.

ACTINB	ATP5A1	CASP3	CDH2	GADD45G	SLUG
TWIST1	BCL2L11	CASP7	CASP8	NANOG	SNAIL
ADM	BIRC3	CASP9	CFLAR	NOL3	TP53
APAF1	BMI1	CDC37	FASLG	POU5F1	XIAP

**Table 2:** The total phenolic and flavonoid content and antioxidant activity of *Vitex agnus-castus* extract.

	*Vitex agnus-castus* Seed Extract
DPPH (IC_50_ µg/mL)	68.19 ± 4.68
ABTS (mg/100 g BHT)	323.529 ± 0.33
Total Phenolic Content (mg/100 g GAE)	1700.77 ± 163.37
Total Flavonoid Content (mg/100 g RE)	5765.86 ± 74.35

## Data Availability

All data supporting the findings of this study are included within the article. Raw data are available from the corresponding author upon reasonable request.

## Abbreviations

The following abbreviations are used in this manuscript:

ADM	Adrenomedullin
APAF1	Apoptotic Protease Activating Factor 1
ATP5A1	ATP Synthase F1 Subunit Alpha 1
BCL2L11	BCL2 Like 11
BIRC3	Baculoviral IAP Repeat Containing 3
CASP	Caspase
CDH2	Cadherin 2
CDC37	Cell Division Cycle 37
CFLAR	CASP8 and FADD Like Apoptosis Regulator
ELISA	Enzyme-Linked Immunosorbent Assay
FASLG	Fas Ligand
FBS	Fetal Bovine Serum
GADD45G	Growth Arrest and DNA Damage-Inducible Gamma
MMP	Mitochondrial Membrane Potential
NANOG	Homeobox Transcription Factor Nanog
NOL3	Nucleolar Protein 3
PBS	Phosphate-Buffered Saline
POU5F1	POU Class 5 Homeobox 1
SLUG	Snail Family Transcriptional Repressor 2
SNAIL	Snail Family Transcriptional Repressor 1
TWIST1	Twist Family BHLH Transcription Factor 1
XIAP	X-Linked Inhibitor of Apoptosis Protein
